# Mitigating the Negative Effect of Drought Stress in Oat (*Avena sativa* L.) with Silicon and Sulphur Foliar Fertilization

**DOI:** 10.3390/plants11010030

**Published:** 2021-12-23

**Authors:** Erika Kutasy, Erika Buday-Bódi, István Csaba Virág, Fanni Forgács, Anteneh Agezew Melash, László Zsombik, Attila Nagy, József Csajbók

**Affiliations:** 1 Institute of Crop Sciences, Faculty of Agricultural and Food Sciences and Environmental Management, University of Debrecen, Böszörményi Street 138, H-4032 Debrecen, Hungary; virag.istvan.csaba@agr.unideb.hu (I.C.V.); forfazsu@gmail.com (F.F.); antenehagezew2008@gmail.com (A.A.M.); csj@agr.unideb.hu (J.C.); 2 Institute of Water and Environmental Management, Faculty of Agricultural and Food Sciences and Environmental Management, University of Debrecen, Böszörményi Street 138, H-4032 Debrecen, Hungary; bodi.erika@agr.unideb.hu (E.B.-B.); attilanagy@agr.unideb.hu (A.N.); 3 Institutes for Agricultural Research and Educational Farm, University of Debrecen, Böszörményi Street 138, H-4032 Debrecen, Hungary; zsombik@agr.unideb.hu

**Keywords:** silicon, sulphur, winter oat varieties, photosynthesis, carotenoid content, water use efficiency, drought

## Abstract

A field experiment was carried out in the 2020–2021 growing season, aiming at investigating the abiotic stress tolerance of oat (*Avena sativa* L.) with silicon and sulphur foliar fertilization treatments and monitoring the effect of treatments on the physiology, production and stress tolerance of winter oat varieties. In the Hungarian national list of varieties, six winter oat varieties were registered in 2020, and all of the registered varieties were sown in a small plot field experiment in Debrecen, Hungary. The drought tolerance of the oat could be tested, because June was very dry in 2021; the rainfall that month totaled 6 mm only despite a 30-year average of 66.5 mm, and the average temperature for the month was 3.2 °C higher than the 30-year average. Foliar application of silicon and sulphur fertilizers caused differences in the photosynthesis rate, total conductance to CO_2_, transpiration, water use efficiency, leaf area, chlorophyll content, carotenoid content, thousand kernel weight (TKW) and yield of winter oat. The application of silicon significantly increased the photosynthesis rate (16.8–149.3%), transpiration (5.4–5.6%), air–leaf temperature difference (16.2–43.2%), chlorophyll (1.0%) and carotenoid (2.5%) content. The yield increased by 10.2% (Si) and 8.0% (Si plus S), and the TKW by 3.3% (Si) and 5.0% (Si plus S), compared to the control plots. The plants in the control plots assimilated less CO_2_ while transpiring 1 m^3^ water more than in the Si, S or Si plus S fertilized plots. The effect of the silicon varied from 9.0 to 195.4% in water use efficiency (WUE) in the three development stages (BBCH52, BBCH65 and BBCH77). A lower leaf area index was measured in the foliar fertilized plots; even so, the yield was higher, compared to that from the control plots. Great variation was found in response to the foliar Si and S fertilization among winter oat varieties—in WUE, 2.0–43.1%; in total conductance to CO_2_, 4.9–37.3%; in leaf area, 1.6–34.1%. Despite the droughty weather of June, the winter oat varieties produced a high yield. The highest yield was in ‘GK Arany’ (7015.7 kg ha^−1^), which was 23.8% more than the lowest yield (‘Mv Kincsem’, 5665.6 kg ha ^−1^). In the average of the treatments, the TKW increased from 23.9 to 33.9 g (41.8%). ‘Mv Hópehely’ had the highest TKW. Our results provide information about the abiotic stress tolerance of winter oat, which, besides being a good model plant because of its drought resistance, is an important human food and animal feed.

## 1. Introduction

Oat (*Avena sativa* L.) is an important annual crop produced on a global scale as human food and animal feed [1]. Its grain is renowned as a rich source of dietary fiber, such as soluble fiber β-glucan, as well as being high in antioxidants, minerals, and vitamins [2]. Nevertheless, as compared with wheat, rice, and barley, oat is undervalued, despite its unique composition that includes many of the nutrients essential for human health and a reduced risk of degenerative disease incidences [3]. It is worth mentioning that oat is a versatile crop that can be grown under marginal environmental conditions, including cool wet climates and unfertile or arid areas [4]. This variation is more pronounced at the genetic level since winter oat showed a 25–30% grain, biomass, and straw yield advantage over the spring oat varieties [5]. However, in most countries, the cultivated area and production volume of oats have progressively declined in the past decades [3,6], even though the demand for oat grain for human consumption has gradually increased, probably due to the dietary or nutraceutical benefits of whole grain [7]. This implies that there is an enormous gap between oat grain supply and demand.

The wider gap in supply and demand of oat grain could be attributed to a complex interaction of several biotic and abiotic factors. Indeed, the productivity, quality, and physiology of oat, as in all cereals, have been observed to be affected by multiple production factors such as nutrient supply, genetic landscape, and environmental variability [3]. Hence, if the agricultural sector is to provide adequate food for the rapidly increasing world population, crops providing a significant complement of human nutrition and energy demand, such as oats, must be responsive to the applied agro-technical measures, particularly for nutrients. A huge varietal difference in response to nutrient application has been observed in a range of oat varieties [8,9]. The grain yield, physiology, and quality of oat have been significantly dictated by the genetic makeup of varieties and their reaction to the environment [6,10]. Allwood et al. [2] verified that selection of suitable oat varieties is equally as important as nutrient application, as far as grain yield is considered.

The integrated use of macronutrients and mineral fertilizers for tackling soil fertility depletion and sustainably improving yields by enhancing the physiological efficiency of oat has paramount importance. Long-term intensive application of chemical fertilizers decreases the available silicon content of the soil; silicon could become a limiting factor in producing high yields, especially in silicon-accumulating crops [11,12]. There is convincing evidence that the application of silicon-containing fertilizers increases grain yield in most cereal crops [13] by improving their lodging, disease, and pest resistance [14,15,16]. It has been estimated that silicon application in the form of fertilizers during the growth period could increase the grain yield of oat by about 34% [17]. Most yield-attributed traits such as the number of seeds spike^−1^, thousand-seed weight, and spike density have been improved after silicon fertilization, which was later reflected in the grain yield of wheat [18,19]. This is partly due to silicon’s important physiological role in promoting plant growth by improving photosynthesis efficiency [20,21] and plant tolerance to various stresses [22,23,24]. Nevertheless, to the best of our knowledge, there has been a noteworthy lack of study and information on silicon fertilization and its effect on grain yield, physiological components, and quality in winter oat varieties.

The other most important nutrient that determines crop yield is sulphur. It is recognized that the application of sulphur during the crop growing period increases oat grain yield and quality [25]. At the physiological level, sulphur enhances the leaf area index (LAI), which boosts intercepted photosynthetically active radiation (IPAR), radiation use efficiency (RUE), and consequently the biomass and grain yield [26]. The grain yield is often determined by the accumulation of more biomass and its partitioning [27,28]. Biomass production is most dependent on the capacity of the canopy (a) to utilize IPAR as a function of LAI, and (b) to convert the intercepted radiation into new biomass, i.e., radiation use efficiency [29,30]. However, a reduction in leaf area index and photosynthetically active radiation has been observed under sulphur deficit conditions [26]. Hence, the positive effects of sulphur and silicon on physiology, yield formation, nutrient uptake, and grain quality of oat substantiate the need to include these essential nutrients in the cultivation system of this species.

Global climate change is expected to increase the frequency of extreme weather such as the number of very hot days, low precipitation, water scarcity, and, after long dry periods, very intensive rain [31,32]. Droughts can have a strong effect on agricultural production, decreasing the yield and increasing yield fluctuations [33].

As silicon and sulphur are known to have numerous beneficial effects on plant growth, development, and productivity and alleviate the negative effects of diverse abiotic stress (e.g., drought), our hypothesis was that foliar silicon (potassium silicate) and sulphur fertilization could be a potential method to mitigate the negative effects of drought on winter oat production.

The objective was to examine the impact of Si and S and their combined application in field experiments and determine whether their application could be an effective and practical method of achieving sustainable oat production. Furthermore, we sought to shed light on varietal differences in the response of registered Hungarian varieties and identify the most productive variety for a given agro-ecological region. Therefore, the photosynthesis rate, total conductance to CO_2_, transpiration, water use efficiency, leaf area, chlorophyll content, carotenoid content, thousand kernel weight (TKW), and yield were analyzed.

## 2. Results

In this study, the parameters of winter oat varieties were analyzed on the basis of a wide range of field measurements in three developmental stages (BBCH52, BBCH65, BBCH77) of the growing season. Relationships among the recorded or calculated parameters were also analyzed.

### 2.1. Evaluation of Water Status for Winter Oat in the Experiment Site

**Figure 1:**
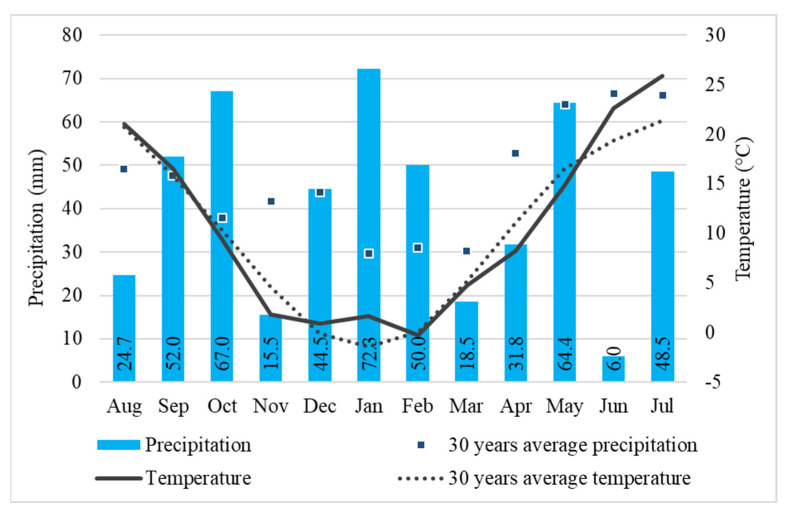
Monthly precipitation totals and average temperatures compared to the 30-year average (Debrecen, 2020/2021). Numbers in the bars: total precipitation for the month. 30-year average: monthly averages for the period 1981–2010.

Climatic conditions are typical continental with cold winters, hot and dry summers, and droughty periods. Figure 1 shows the climatic conditions during the 2020/2021 crop year for the experiment in Debrecen. Due to climate change, the average temperature from August 2020 to July 2021 was 0.3 °C higher (10.6 °C), than the 30-year (1981–2010) average (10.3 °C). The effect of climate change was more pronounced in winter; the average temperature in December, January, and February (2020/2021) was 0.8 °C, while the 30-year average for Debrecen was −0.5 °C. The mild winter did not allow testing the cold stress tolerance of the winter oat varieties and the effect of the treatments on hardiness. On the contrary, because it was dry, the growing season offered the opportunity to test the drought stress tolerance of the varieties and the effect of the treatments on changing water use. Total precipitation from September 2020 to June 2021 was 422 mm, lower than the 30-year average of 444.9 mm for the same period, and the distribution was uneven. November, March, April, and June were very dry months. In the period from March to June, total rainfall was 92.8 mm less than the 30-year average. According to climate change model predictions, it is expected that Hungary will experience more severe drought events, while on the other hand, more extreme precipitation events in the future are also foreseen (Juhász et al. [34]).

June was very dry in 2021; the rainfall totaled 6 mm only in the whole month, whereas the 30-year average is 66.5 mm; moreover, the temperature was very high, averaging 3.2 °C higher than the 30-year average for June. These conditions caused serious water stress to the oat varieties.

**Figure 2:**
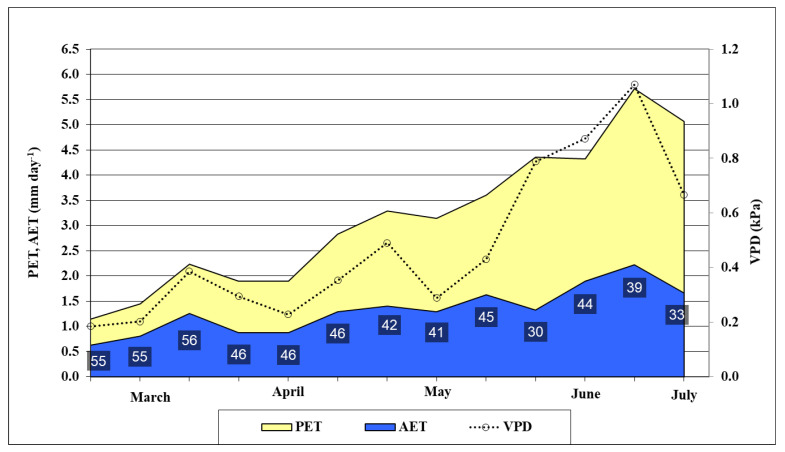
Potential (PET) and actual (AET) evapotranspiration, AET/PET ratio (%), and VPD in oats from March to June (Debrecen, 2021). Average of the decades. VPD: Water pressure deficit, calculated using meteorological data.

**Figure 3:**
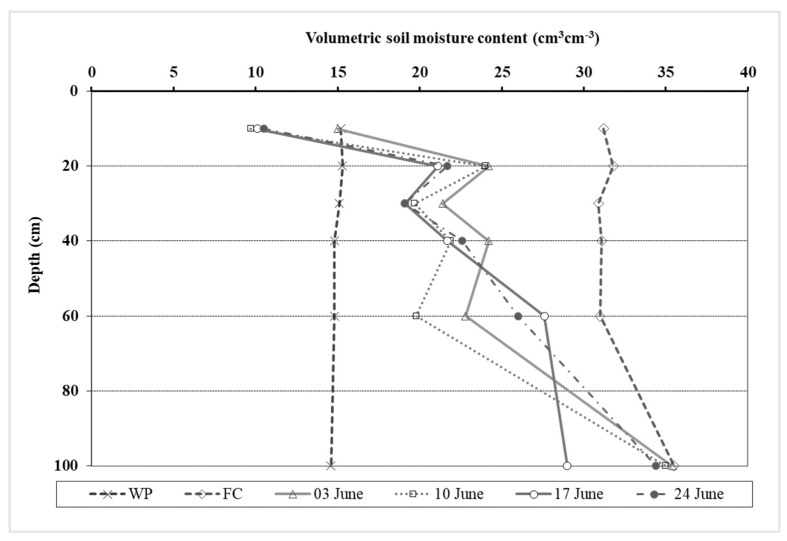
Soil moisture content in the 0–100 cm layer in the oat experiment from 3 to 24 June. (Debrecen, 2021). WP: permanent wilting point; FC: field capacity.

The potential and actual evapotranspiration (PET and AET), their ratio, and the vapor potential deficit (VPD) of the air were calculated to characterize the water status for oat (Figure 2). Evapotranspiration data supported that there was a very dry growing season in 2021. From the first decade of March to the last decade of June, the AET was only 45% of the PET value on average. The soil was very dry in spring despite the abundant precipitation of January and February. The AET/PET ratio was low even in March (55–56%). In April and May, the ratio varied between 41 and 46%. These low values show that the evapotranspiration was limited by the very low available moisture content in the root zone in the soil. In June, long droughty periods were recorded without rainfall and the oat was in serious water stress. The AET/PET ratio was between 30–44%. The VPD value calculated using the meteorological data collected at the experiment site also backed this statement, since it varied between 0.79 and 1.07 in June. The recorded soil moisture content data also supported that the oat plants were in water stress in June. The moisture content of the soil decreased and was very low, especially in the upper 60 cm layer. At 10 cm depth, the soil was drier (9.7–15 V%) than the permanent wilting point (15.2 V%). The water deficit varied between 110.4–122.9 mm in the 0–60 cm layer in June (Figure 3).

### 2.2. Response of Winter Oat Varieties to Drought Stress and the Effect of Foliar Fertilization through the Photosynthesis Parameters and Transpiration

**Figure 4:**
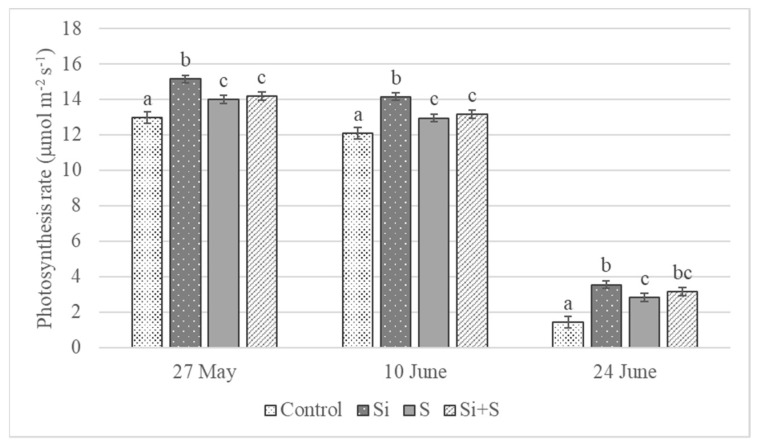
Assimilation rate of winter oat, (Debrecen, 2021), mean ± SE of three replicates. Different lower-case letters (a–c) mean significant difference at *p* < 0.05 among the treatments. When the letters on the different treatments are the same, it means there is no significant difference between them.

**Figure 5:**
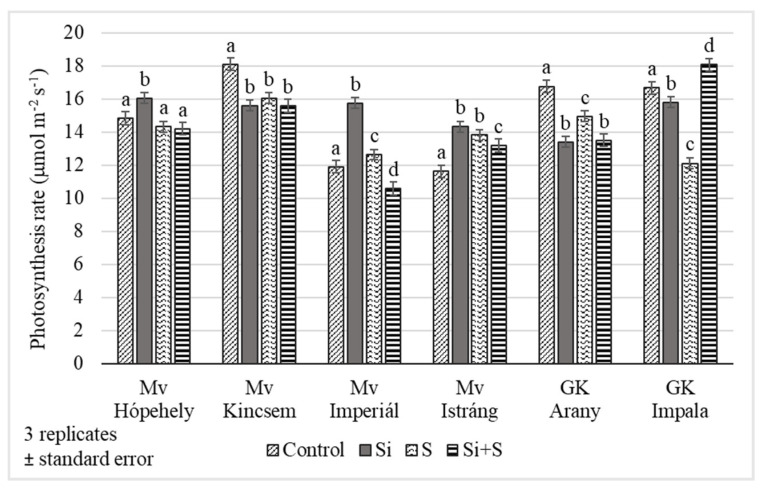
Assimilation rate of winter oat varieties, (Debrecen, 27 May, BBCH52), standard error of means. The different lower-case letters (a–d) mean significant difference at *p* < 0.05 among the treatments in the varieties. When the letters on the different treatments are the same, it means there is no significant difference between them.

The foliar fertilization treatments resulted in changes in the assimilation rate of winter oat during the growing season. The differences were significant on every date, *p* = 0.023 on 27 May, *p* < 0.001 on 10 and 24 June. The highest values were measured on 27 May (BBCH 52) (Figure 4). The lowest assimilation rates were observed in control plots (12.98 μmol m^−2^ s^−1^) and the highest in the Si fertilized plots (15.15 μmol m^−2^ s^−1^) on average, but the varieties’ reactions varied. The photosynthesis rate differed significantly between the Si fertilized and control plots in every variety. In the ‘Mv Hópehely’, ‘Mv Imperiál’ and ‘Mv Istráng’ varieties, the highest CO_2_ assimilation rate was observed under Si fertilization treatment, while in the ‘Mv Kincsem’ and ‘GK Arany’, the control plots produced the highest assimilation values (Figure 5), so the response of these varieties to the treatments differed. The average difference for the varieties was not significant between the S and Si plus S fertilized plots.

**Figure 6:**
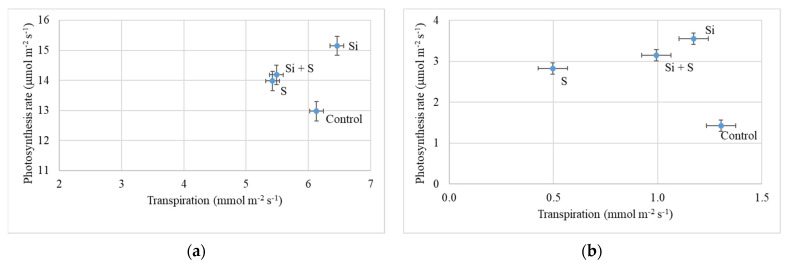
(**a**) Photosynthesis rate of oat as a function of transpiration (27 May, BBCH52); (**b**) Photosynthesis rate of oat in the function of transpiration (24 June, BBCH77). Means of the varieties, ± standard error.

Figure 6 shows the relationship between photosynthesis and transpiration on 27 May (BBCH52) and 24 June (BBCH77). The effect of the foliar fertilization treatments was similar, but in June both the transpiration and photosynthesis rates were much lower than in May. Si fertilized plants had a high assimilation rate (15.15 μmol m^−2^ s^−1^) in addition to high transpiration (6.46 mmol m^−2^ s^−1^) compared to the other treatments. In the control plots, the plants had high transpiration (6.13 mmol m^−2^ s^−1^), but the lowest level of photosynthesis (12.98 μmol m^−2^ s^−1^). Sulphur treated plots showed the lowest transpiration (5.42 mmol m^−2^ s^−1^), and the difference was more pronounced on 24 June. The differences in photosynthesis rate and transpiration were significant at all three measurement times among the treatments (*p* < 0.001).

**Figure 7:**
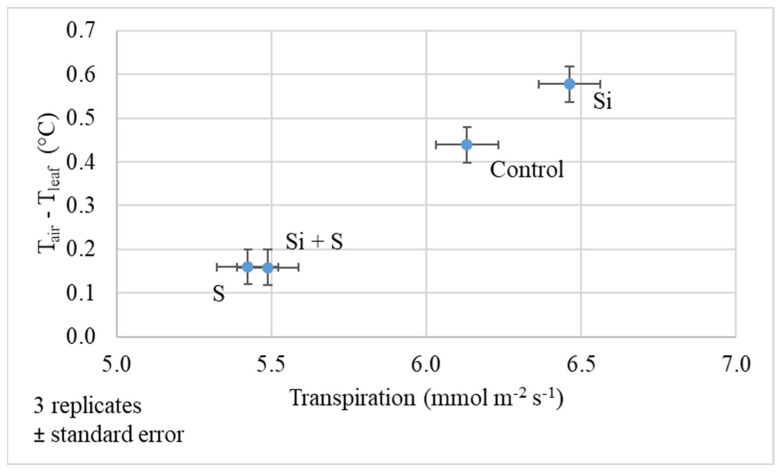
The difference in air and leaf temperature of oat as a function of transpiration (27 May, BBCH52); Means of the varieties, ± standard error.

The difference between the air and leaf temperature gives information about the water status of the plants. The higher the difference, the better the cooling effect of transpiration. In water stress, because the stomata of the leaf are closing, the cooling effect is low. Figure 7 shows that on 27 May the oat had a relatively good water status, and the transpiration was at a normal level. Si fertilized plants produced the highest transpiration (6.46 mmol m^−2^ s^−1^) and air–leaf temperature difference (0.58 °C). The changes were significant among the treatments both in transpiration and air–leaf difference (*p* < 0.001). The leaf cooling effect was lower in S and S plus Si fertilized plots (0.16 °C).

**Figure 8:**
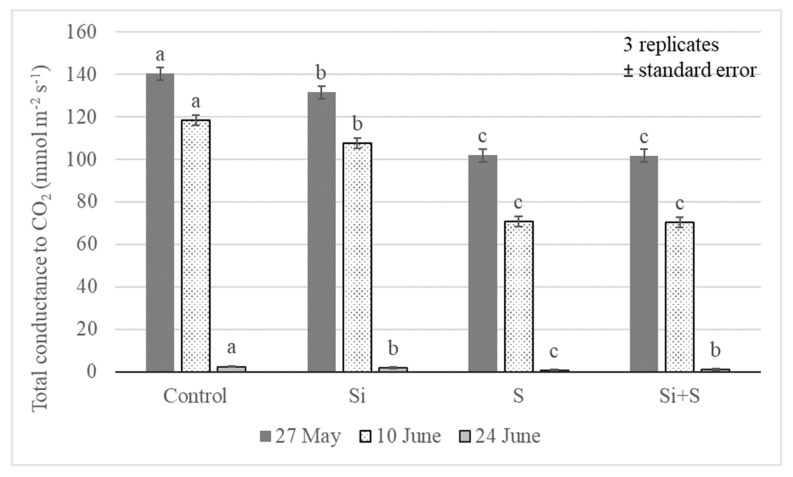
Total conductance to CO_2_ (GTC) in oat leaves as an effect of the treatments (Debrecen, 2021); Means of the varieties, ± standard error. The differences among the treatments were significant at *p* = 0.01. The different lower-case letters (a–c) represent significant difference at *p* < 0.05 among the treatments. When the letters on the different treatments are the same, it means there is no significant difference between them.

Total conductance to CO_2_ (GTC) of the leaves is a combination of resistances of the boundary layer and stomata on both sides of the leaf against CO_2_ diffusion. In our experiment, the treatments induced significant differences in the GTC value for the winter oat leaves at *p* = 0.01 level. The highest GTC values were on 27 May (BBCH52); afterward, the GTC followed the decrease in soil moisture content in the root zone (Figure 8) in every treatment. The highest GTC was observed in the control plots at all three measurement times: 140.3, 118.4, and 2.4 mmol m^−2^ s^−1^, respectively. There were no significant differences between the S and Si plus S fertilized plots in BBCH52 and BBCH65 development stages. The last measurement showed very low values for GTC (2.4, 1.7, 0.6, 1.1 mmol m^−2^ s^−1^). The reasons were probably the very dry soil in June, causing water stress, and the start of senescence in the plants.

The correlations between the assimilation and water use parameters were inspected (Table 1). We expected to see a close relationship between total conductance and some assimilation values because stomata opening affects them. The correlation coefficients pointed to a very strong positive connection between the photosynthesis rate and transpiration (*r* = 0.888), the photosynthesis rate and GSW (stomatal conductance to water vapor) (*r* = 0.876), and the GSW and transpiration (*r* = 0.937). The correlation was strong but negative between the GSW and VPD_leaf_ (*r* = −0.873), the GSW and T_leaf_-T_air_ (*r* = −0.801), and transpiration and T_leaf_-T_air_ (*r* = −0.789). The analysis proved a connection between intercellular CO_2_ level and WUE (*r* = −0.745), photosynthesis rate and VPD_leaf_ (*r* = −0.698), and transpiration and VPD_leaf_ (*r* = −0.706).

### 2.3. Effects of the Treatments on the Chlorophyll and Carotenoid Content of Oat

**Figure 9:**
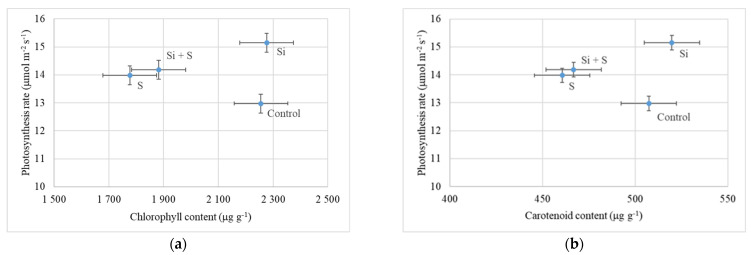
(**a**) Photosynthesis rate of oat as a function of chlorophyll content (27 May, BBCH52); (**b**) Photosynthesis rate of oat as a function of carotenoid content (24 June, BBCH77). Means of the varieties, ± standard error.

There were no significant differences among either the varieties or the treatments in chlorophyll content (*p* = 0.63 and 0.456), but the control and Si fertilized plants had higher chlorophyll content. The chlorophyll content of the leaves was closely corelated with the photosynthesis rate. The higher chlorophyll content resulted in a higher CO_2_ assimilation rate in the fertilized plots of our experiment (Figure 9a). The control plots showed lower photosynthesis (12.98 μmol m^−2^ s^−1^) and high chlorophyll content (2255.1 μg g^−1^). Si fertilized plants also had high chlorophyll content (2276.41 μg g^−1^), but also a high level of photosynthesis (15.15 μmol m^−2^ s^−1^). Similar results were observed in total carotenoid content; the differences were also not significant, but the control and Si fertilized plants had higher carotenoid content (Figure 9b).

The relation was examined also between the carotenoid content and the photosynthesis rate. In the Si fertilized plots, the higher carotenoid content (519.8 μg g^−1^) resulted in a higher assimilation rate (Figure 9b). In the control plots, the relatively high carotenoid content (507.4 μg g^−1^) was accompanied by a lower level of photosynthesis. Close correlation was found between the chlorophyll and carotenoid content of the plants (r = 0.892; *p* = 0.01).

**Figure 10:**
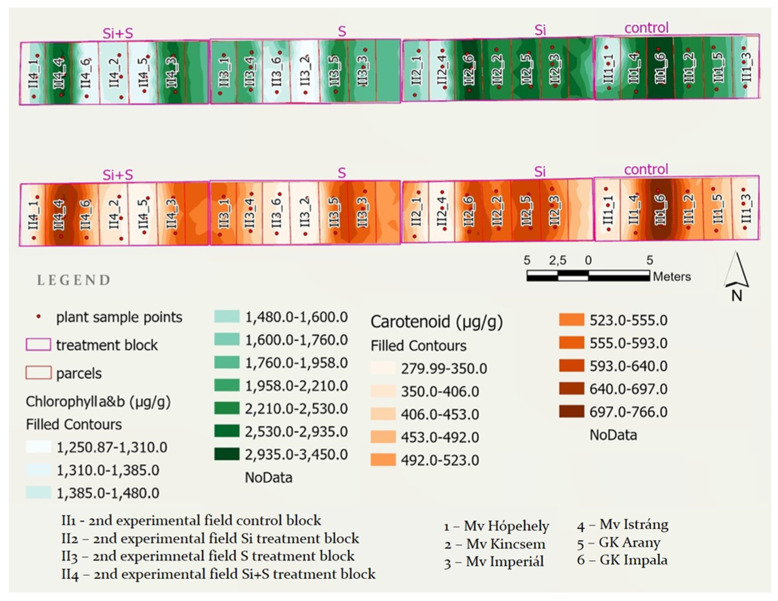
Chlorophyll a and b and carotenoid content spatial distribution maps generated in ArcGIS Pro from point sample laboratory measurements.

To visualize chlorophyll a and b and carotenoid values for the examined oat varieties, two maps were created showing the four treatment blocks of the middle experiment with the parcels and sample points within. Both maps were classified using the geometrical interval classification method provided by ArcGIS Pro (Figure 10).

### 2.4. Water Use Efficiency of Winter Oat Varieties

The water use efficiency (WUE) was calculated using the assimilated CO_2_ and transpirated H_2_O values measured by the Li-6800 portable photosynthesis system (Table 2). The treatments yielded statistically significant differences in oat WUE on all three dates (*p* < 0.001). The plants in the control plots assimilated less CO_2_ while they transpirated 1 m^3^ more water than the other plots every time; the differences varied from 9.0% to 19.8% on 27 May (BBCH52), from 15.1% to 44.0% on 10 June (BBCH65), and from 195.4% to 272.0% on 24 June (BBCH77).

The differences in WUE were also significant among the varieties (*p* < 0.001). The differences varied from 2.0% to 9.9% on 27 May (BBCH52), from 4.6% to 24.0% on 10 June (BBCH65), and from 6.3% to 43.1% on 24 June (BBCH77) (Table 3).

### 2.5. Leaf Area Changes as an Effect of the Treatments

**Figure 11:**
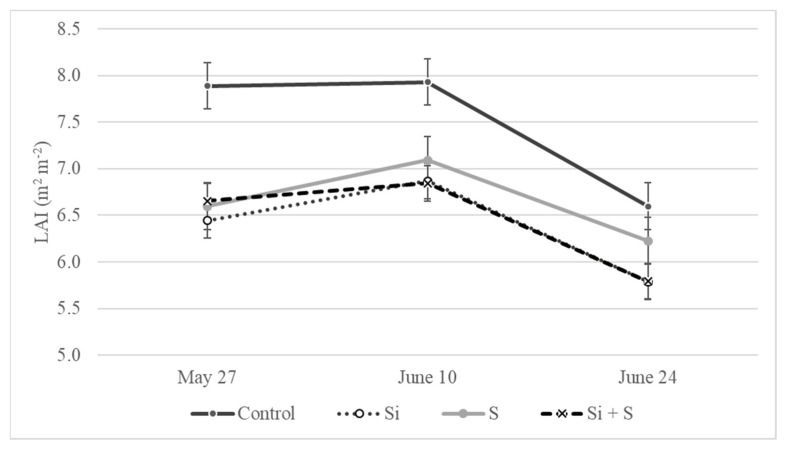
LAI values for winter oat as an effect of the treatments (Debrecen, 2021); Means of the varieties, ± standard error. The differences among the treatments were significant at *p* = 0.05.

The leaf area index data were measured three times during the growing season, in parallel with the other measurements (BBCH52, BBCH65, and BBCH77). Effective foliar fertilization requires a high leaf area index for absorbing applied nutrient solutions in sufficient amounts. Due to good soil conditions, strong root systems, and favorable weather conditions in May, the oat plants developed large leaf area. The highest LAI values were recorded on average during the BBCH65 stage (10 June) of the varieties in every treatment (7.9, 6.9, 7.1, 6.9 m^2^ m^−2^). Only the ‘Mv Istráng’ and ‘GK Arany’ varieties showed the highest leaf area earlier, during the BBCH52 stage (27 May). Statistical analysis showed that the differences were not significant among the varieties on 27 May and 24 June (*p* = 0.78 and 0.40), but were statistically proved on 10 June (*p* = 0.004). ‘Mv Impala’ and ‘Mv Imperiál’ developed the highest leaf area among the varieties, as high as 7.98 and 7.93 m^2^ m^−2^. The effect of the foliar fertilization treatments on the LAI was significant at all three time points (*p* = 0.002, 0.011, and 0.015, respectively). The oat plants developed a larger leaf area in the control plots compared to the other treatments (Figure 11). The differences were between 0.81 and 1.44 m^2^ m^−2^. Leaf area index values for the Si and Si plus S fertilized plots were the lowest and did not diverge from each other.

### 2.6. Evaluation of the Yield and TKW of the Winter Oat Varieties

Despite the very dry weather conditions in June, the winter oat varieties produced a high yield in the experiment (Table 4). The mean yield of the experiment was 6374.4 kg ha^−1^. Significant differences were observed in the yield and TKW among the varieties (*p* < 0.001). On average, for the treatments, the highest yield was harvested in ‘GK Arany’ (7015.7 kg ha^−1^), which was 23.8% more than the lowest yield (‘Mv Kincsem’, 5665.6 kg ha ^−1^). The thousand kernel weight varied relatively greatly among the varieties. The average for the treatments ranged from 23.9 to 33.9 g (41.8%). ‘Mv Hópehely’ had the highest TKW, and ‘GK Impala’ had the lowest value.

Furthermore, the foliar fertilization treatments also resulted in statistically proven differences in the yield and TKW at *p* = 0.010 and *p* = 0.015 levels, respectively. The control plots gave the lowest yield (6004.1 kg ha^−1^). Si treatment resulted in the highest yield (6615.3 kg ha^−1^) on average for the varieties. This was 10.2% higher than the control plots’ yield. The surplus yield in the Si plus S fertilized plots was 8.0%, and in the S fertilized plots, 6.5%, compared to the control plots. On average for the varieties, the thousand kernel weight values for the control, Si, S, and Si plus S treatments were 29.9 g, 30.9 g, 28.8 g, and 31.4 g, respectively. The Si plus S treatment resulted in the highest TKW.

The varieties reacted differently to the foliar fertilization, but the interaction was not significant. ‘GK Arany’ produced the highest yield in all the foliar fertilized plots, followed by ‘Mv Istráng’ and ‘Mv Imperiál’ in the Si fertilized plots, ‘Mv Istráng’ and ‘GK Impala’ in the Si plus S treatment, and ‘Mv Hópehely’ in the S treatment.

## 3. Discussion

Potassium silicate fertilization provides an excellent source of soluble silicon for crops and contributes also to the potassium status in the plants [35].

In our experiment, foliar silicon and sulphur fertilizer applications caused differences in the CO_2_ assimilation parameters, transpiration, water use efficiency, leaf area, chlorophyll content, carotenoid content, TKW, and yield of winter oat. Silicon fertilization significantly increased the photosynthesis rate, transpiration, air-leaf temperature difference, chlorophyll, and carotenoid content. Similar observations were reported by other researchers. Botta et al. [15] and Dinesh et al. [36] reported that silicon-containing fertilizer application increased the yield of most cereal crops. Sorrato et al. [17] also reported similar findings in oat; they found that silicon application increased the thousand–seed weight, the number of seeds per spike, and, in consequence, the yield, by about 34%. Our results are consistent with these results, but the effect was lower; the yield increased by 10.2% (Si) and 8.0% (Si plus S). Silicon application also increased the TKW in our experiment, but only by 3.3% (Si) and 5.0% (Si plus S), compared to the control.

Foliar fertilization changed the water use efficiency (WUE) of oat in our experiment. The plants in the control plots assimilated less CO_2_ but transpirated 1 m^3^ more water than the Si, S, or Si plus S fertilized plants. The treatments reduced the water stress effect on the oat plants’ physiological functions. The effect of the silicon varied from 10.8 to 63.9% in WUE in the three development stages (BBCH52, BBCH65, and BBCH77). Other researchers reported similar observations in oat, wheat, and barley. Ahmad et al. [23], Frew et al. [37], and Ciecierski [38] proved the positive effect of silicon application in reducing the negative impact of drought stress and increasing the yield of wheat. Bocharnikova et al. [39] also reported similar results with their research, which sought to determine the effect of silicon fertilizer on the drought resistance of barley. Findings by Farkas et al. [40] based on silicon application research in the winter oat variety ‘Mv Hópehely’, also supports our findings regarding the positive effect of Si fertilization on water use efficiency.

The foliar fertilization treatments had a significant effect on the leaf area. The control plots had a larger leaf area compared to the other treatments. Leaf area index values of the Si and Si plus S fertilized plots were the lowest and did not diverge from each other. This observation is in contrast with the findings of Salvagiotti and Miralles [26]. They reported that sulphur application enhanced the leaf area of wheat. Based on our results, we can state that oat with lower leaf area produced higher yield in the foliar fertilized plots; therefore, the increased photosynthesis rate not only compensated for the decreased LAI, but was also able to produce surplus yield. Smaller leaves denote an advantage in a hot and dry environment with high solar radiation, as an adaptation to the dry conditions [41]. It seems that the application of Si and Si plus S fertilizers can help oat plants adapt to drought-like conditions by inducing smaller leaf area development.

The surplus yield in the Si plus S fertilized plots was 8.0%, and in the S fertilized plots, 6.5%, compared to that in the control plots. Pandey’s [42] results for oat, in correlation with our findings, showed that the yield from sulphur application, in addition to the balanced use of NPK nutrients, outperformed the yield with NPK fertilizers alone, by 7.1%. Kurmanbayeva et al. [43] in a greenhouse setting and Klikocka et al. [44] in a field experiment also observed similar increases (3.58%) in grain yield with sulphur fertilization in wheat.

The cooling effect of transpiration in decreasing leaf temperature is very important for plants; the chemical and biological processes inside the leaf depend on temperature. Foliar application of sulphur also changed the water management of the winter oat varieties significantly. Changes in leaf temperature caused changes in transpiration at constant VPD. In the sulphur fertilized oats, transpiration was lower and the photosynthesis rate was reduced, but the decrease in assimilation was also lower, resulting in higher water use efficiency—the best among the treatments.

The application of silicon fertilizers increased the total chlorophyll content, carotenoid content, and the photosynthesis rate of the treated oats. Our observations are supported by other results [45,46].

Large variations were observed in response to the foliar Si and S fertilization treatments among the winter oat varieties: 2.0–43.1% in WUE, 4.9–37.3% in total conductance to CO_2_, and 1.6–34.1% in leaf area. The increased photosynthetic activity and water use efficiency of the canopy increased carbohydrate production, resulting in greater yield and eventually contributing to better yield quality [47].

We plan to test other silicon fertilizers and forms without potassium content, in order to isolate the effects of silicon specifically.

## 4. Materials and Methods

### 4.1. Soil Characteristics of the Experimental Site

The experiment was set up in fall 2020 at the research site of Debrecen University, Hungary; the coordinates are 47°33′02″ N; 21°35′56″ E. The area has homogeneous chernozem soil; in the World Reference Base for Soil Resources, it is Calcic Endofluvic Chernozem (Endosceletic) [48].

The humus content of the upper layer is good (Hu% = 2.7–3.66), the thickness of the humus layer is around 80 cm. The soil plasticity index (K_A_) is 38. The acidity of the upper soil layers is slightly alkaline (pH_H2O_ = 8.3–8.43). The phosphorus status of the calcareous soil is very good (AL-soluble P_2_O_5_ 1076.8–1671.6 mg kg^−1^), and its potassium status is also very good (AL-soluble K_2_O 525.5–658.9 mg kg^−1^). (Table 5). The soil has a favorable water regime, and the water table is at 6–7 m depth.

### 4.2. Experimental Setup

The measurements were carried out in a small plot (1.5 m × 7 m = 10.5 m^2^) experiment, with three independent repetitions. Sowing was performed on 26 October 2020, with 550 seeds per m^2^ seed rate at a depth of 5 cm, and the crop was harvested on 9 July 2021.

The six tested winter oat (*Avena sativa L.)* varieties—‘Mv Hópehely’, ‘Mv Kincsem’, ‘Mv Imperiál’, ‘Mv Istráng’, ‘GK Arany’, and ‘GK Impala’—are locally bred, new, and promising varieties. ‘Mv Hópehely’ is the first (2007) winter oat variety of Agricultural Institute, Centre for Agricultural Research, Eötvös Loránd Research Network, Martonvásár, Hungary, while ‘Mv Kincsem’, ‘Mv Imperiál’, and ‘Mv Istráng’ were registered in 2016. ‘Mv Imperiál’ is the first black-seeded winter oat in Hungary. ‘GK Arany’ (2017) and ‘GK Impala’ (2005) are the varieties of Cereal Research Non-Profit Ltd., Szeged, Hungary.

Fertilization was with N_20_P_40_K_120_ kg ha^−1^ in October 2020 and N_50_ kg ha^−1^ on 26 February 2021.

We applied 4 treatments:

1. Control, without foliar fertilization
2. Silicon fertilization (Si) 3.0 L ha^−1^
3. Sulphur fertilization (S) 5.0 L ha^−1^
4. Silicon plus sulphur fertilization (Si + S) 3.0 + 5.0 L ha^−1^

Foliar fertilizers:

- Sulphur fertilizer: liquid foliar fertilizer with high sulphur content (lignosulfonate formulation) 1000 g L^−1^ SO_3_, 30 g L^−1^ N, 30 g L^−1^ MgO, 27 g L^−1^ B, 0.003 g L^−1^ Mo
- Silicon fertilizer: (potassium silicate formulation) 1.4 m/m% Si, 10.5 m/m% K_2_O

Foliar fertilization application times:

1 December 2020 BBCH13 (3 leaves unfolded)
10 May 2021   BBCH39 (flag leaf stage)
18 June 2021   BBCH73 (early milk)

### 4.3. Measurements, Calculations and, Their Methodology

We measured the photosynthesis parameters, release of water vapor (H_2_O) by leaf, and leaf area index (LAI) 3 times (27 May (BBCH 52); 10 June (BBCH 65); 24 June (BBCH 77) [49], as well as the maximum plant height, grain yield, and grain moisture content.

Photosynthesis parameters and transpiration were measured in intact leaves using the LI-6800 (LI-COR, Lincoln, NE, USA) portable photosynthesis system. It has two high-precision infrared gas analyzers to measure CO_2_ and H_2_O mole fraction in air. Using input and output of CO_2_ (μmol mol^−1^) and H_2_O (mmol mol^−1^), leaf temperature (°C), atmospheric pressure (kPa), flow rate (μmol s^−1^) and other measured parameters, the instrument calculates net assimilation, transpiration, total conductance to CO_2_ (GTC), intercellular CO_2_ concentration [50], and other physiological parameters. The light was controlled in the sample chamber at 1500 µmol photon m^−2^ s^−1^ PAR and set at 90% red (625 nm) and 10% blue (475 nm).

The Li-6800-01A chamber head was used as a light source; the aperture was 2 cm^2^. The CO_2_ concentration was controlled in the chamber at 400 μmol mol^−1^ using injector and carbon-dioxide patrons. The average ambient CO_2_ level was 399.984 μmol mol^−1^. The two leaf thermocouple thermometers in the leaf chamber recorded the leaf temperature during the measurements via contact with the lower leaf surface [51]. Light-adapted leaves were measured, six times per leaf on two plants per plot. Readings were logged when the measured parameters stabilized but after a minimum of 120 s [51]. Air temperature was measured by the LI-6800 in the chamber.

Water use efficiency was calculated from the measured data on the leaves, applying the formula proposed by Tanner and Sinclair [52], (Equation (1)).

(1) $$WUE=\frac{Ass*44}{Emm*18}$$

*WUE*: water use efficiency (kg m^−3^)
*Emm*: transpirated H_2_O (mmol m^2^ s^−1^)
*Ass*: assimilated CO_2_ (μmol m^2^ s^−1^)

The FAO Penman–Monteith method was used in the calculation of potential evapotranspiration (PET) with climatic data collected at the experiment site [53] (Equation (2)).

(2) $$PET=\frac{0.408\Delta \left(R_{n}-G\right)+\gamma \frac{900}{T+273}u_{2}\left(e_{s}-e_{a}\right)}{\Delta +\gamma \left(1+0.34u_{2}\right)}$$

*PET*: potential evapotranspiration (mm day^−1^),
*R_n_*: net radiation at the crop surface (MJ m^−2^ day^−1^),
*G*: soil heat flux density (MJ m^−2^ day^−1^),
*T*: mean daily air temperature at 2 m height (°C),
*u*_2_: wind speed at 2 m height (m s^−1^),
*e_s_*: saturation vapor pressure (kPa),
*e_a_*: actual vapor pressure (kPa),
Δ: slope vapor pressure curve (kPa °C^−1^),
*γ*: psychrometric constant (kPa °C^−1^).

Actual evapotranspiration (AET) was estimated using the formula of Antal [54]. The method uses the PET, the soil moisture data, and the crop coefficient values (Equations (3) and (4)).

(3) $$AET=\frac{w+b}{1+b}*w*PET$$

*AET*: actual evapotranspiration (mm day^−1^),
*w*: relative soil moisture content in the 0–100 cm layer (mm)
*b*: crop coefficient factor of oat
*PET*: potential evapotranspiration (mm day^−1^)

(4) $$w=\frac{W_{a}-WP}{W_{c-}WP}$$

*w*: relative soil moisture content in the 0–100 cm layer (mm)
*WP*: permanent wilting point of the soil (mm)
*Wc*: field capacity of the soil (mm)

Vapor pressure deficit (*VPD*) in kilopascals was calculated from saturated vapor pressure (*VP_sat_*) and actual vapor pressure (*VP_air_*):

(5) $$VP_{sat}=\frac{610.7\times 10^{\;\frac{7.45*T}{237.3+T}}}{1000}$$

(6) $$VP_{air}=\frac{610.7\times 10^{\;\frac{7.45*T}{237.3+T}}}{1000}*\frac{RH}{100}$$

*VP_sat_*: saturated vapor pressure of the air (kPa),
*T*: air temperature (°C)
*VP_air_*: actual vapor pressure of the air (kPa)

(7) $$VPD=VP_{sat}-VP_{air}$$

Soil moisture content was recorded 4 times using Delta-T PR2/6-SDI-12 profile probe (Delta-T Devices Ltd., Cambridge, UK) with 6 sensors (10, 20, 30, 40, 60, 100 cm depth) connected to the HH2 Moisture meter. It was calibrated to the given soil.

Leaf area index (LAI) was measured using an LAI-2000 Plant Canopy Analyzer (LI-COR, Lincoln, NE, USA) in one sensor mode; above-canopy and below-canopy readings were obtained using the same LAI-2050 optical sensor. Two above- and eight below-canopy readings for each plot were performed. The 8 readings were averaged to each of the sensed plots.

Photosynthesis and LAI measurements were taken from 8 to 10 am each time.

Grain on each plot was harvested by Wintersteiger 125 plot combine with 125-cm cutting width. Grain samples were taken from each plot to determine the grain moisture content and 1000 kernels weight.

The total chlorophyll content and total carotenoid content of the plants were measured, and the data were plotted on GIS maps. Leaf samples were collected on 24 June (BBCH 77). Leaf samples were stored and prepared for the measurement in accordance with Szabó et al. [55] study. Leaves were stored refrigerated at 4 °C and shipped, then processed under laboratory conditions within 6 h. The pigment content of the leaf samples was extracted with 80% acetone and 1 g quartz sand for homogeneity. After extraction, the suspensions were centrifuged at 3000 rev/min for 3 min in Hettich ROTOFIX 32A, and the clean solution was placed into 2.5 mL quartz cuvette. The absorbance of the solution was measured by SECOMAN Anthelie Light II UV-VIS spectrophotometer at 470, 644, and 663 nm wavelengths. Chlorophyll a absorbs light in the red range, while chlorophyll b and carotenoids absorb light in the blue range. The measured values were converted to total chlorophyll values, according to the formula by Droppa et al. [56] (Equation (8)):

(8) $$Chla+b=\left(20.2*A_{644}+8.02*A_{663}\right)*\frac{V}{w}$$

Chla + *b*: chlorophyll a and b content (µg/g)
*V*: volume of extracted plant tissue (mL)
*A*_644_: spectral absorbance value at wavelength of 644 nm (unitless)
*A*_663_: spectral absorbance value at wavelength of 663 nm (unitless)
*w*: weight of fresh plant tissue sample (g)

To calculate the carotenoid content of the samples, the formula of Lichenthaler et al. [57] was applied (Equation (9)):

(9) $$Car=\left(1000*A_{470}-3.27*\left(12.21*A_{663}-2.81*A_{644}\right)-104*\left(20.13*A_{644}-5.03*A_{663}\right)\right)*\frac{V}{w}$$

*Car*: carotenoid content (µg/g)
*V*: volume of extracted plant tissue (mL)
*A*_470_: spectral absorbance value at wavelength of 470 nm (unitless)
*A*_644_: spectral absorbance value at wavelength of 644 nm (unitless)
*A*_663_: spectral absorbance value at wavelength of 663 nm (unitless)
*w*: weight of fresh plant tissue sample (g)

As the first phase of GIS work, corner coordinates of all field units were measured with a high-precision Stonex S9i GNSS Reciever RTK GPS device, from which the polygon layer was generated in ESRI ArcGIS Pro. Analogously, plant sample points were measured and converted to a polypoint GIS layer. The plant samples, three random samples from each plot, were collected on 29 June 2021. The values of chlorophyll a and b and carotenoid content of the laboratory measurements were joined as an attribute table to the polypoint layer. Kriging raster interpolation was run in two turns on the point layer data table, which resulted in chlorophyll a and b and carotenoid content spatial distribution maps.

### 4.4. Data Analysis

Data analysis and evaluation were performed using the IBM SPSS Statistics 22.0 (IBM Corp., Chicago, IL, USA) statistical software package. The univariate GLM model was used to compare the means of the different parameters among the varieties and treatments. The descriptive statistics option was switched on. The prerequisites to analysis of variance (normality, homogenous variances, and independency) were checked on the dependent variables. Normality was inspected using the Kolmogorov–Smirnov test. LSD post hoc tests were conducted for pairwise comparisons of the means. The significance level (alpha) was set to *p* = 0.05. Pearson correlation analysis (2-tailed) was run to reveal the linear relationships among the parameters. Significance and ± standard error were presented in the figures and tables where it was relevant.

## 5. Conclusions

Based on our results, Si and S foliar fertilization could be an effective method of stress alleviation in oat production. This study highlighted that the oat varieties responded differently to the water stress, and the effect of the Si and S foliar fertilization also showed varietal variation.

Although oat varieties have good adaptability and the extent is varied with their genetic landscape, foliar-based fertilization with sulphur and silicon could further boost the assimilation efficiency, production, and productivity under any array of environmental stress conditions such as drought.

Si can alleviate the drought stress of oat by improving the photosynthesis rate and water use efficiency, adjusting the chlorophyll content and stomatal conductance, and regulating transpiration.

Under the given conditions, the ‘GK Arany’ and ‘Mv Istráng’ varieties had the highest yields in the 2020/2021 growing season, the difference between the highest and lowest yields was 23.8%. The yield-increasing effect of silicon fertilization was the highest in ‘Mv Kincsem’ (21.7%).

Relying on the timely application of silicon-based fertilizers would seem to be a promising agronomic strategy to counteract the adverse effects of drought-induced stress, but further research is required to clarify the interactions. Additionally, identifying the best formulation for the delivery of Si will require further evaluation to determine the optimum application schedule and dosage in practice.

Scaling up the potential benefits and assessing the economic feasibility of large-scale silicon application, as well as understanding and underlining the mechanism by which silicon improves oat drought tolerance and its interaction with the environment, are very important.

Therefore, we plan to pursue the thoughtful and focused design of multidisciplinary experiments leading to a better understanding of the role of Si and S in oat development and the enhancement of oat resistance to stress.

## Figures and Tables

**Table 1:** Pearson correlation coefficient values between WUE, VPD, and assimilation parameters in winter oat (2021, Debrecen).

	Ci	GSW	Transp	T_leaf_-T_air_	WUE	VPD_leaf_
Ass	−0.355 **	0.876 **	0.888 **	−0.667 **	0.447 **	−0.698 **
Ci	1	0.440 **	0.328 **	−0.461 **	−0.745 **	−0.548 **
GSW	0.440 **	1	0.937 **	−0.801 **	0.058	−0.873 **
Transp	0.328 **	0.937 **	1	−0.789 **	0.013	−0.706 **
T_leaf_-T_air_	−0.461 **	−0.801 **	−0.789 **	1	0.103 *	0.686 **
WUE	−0.745 **	0.058	0.013	0.103 *	1	−0.108 *

Ass: photosynthesis rate; Ci: intercellular CO_2_ level; GSW: stomatal conductance to water vapor; Transp: transpiration; T_leaf_-T_air_: difference between leaf and air temperature; WUE: water use efficiency; VPD_leaf_: vapor pressure deficit on the leaf. *: the correlation is significant at *p* = 5%; **: the correlation is significant at *p* = 1%.

**Table 2:** Effect of treatments on water use efficiency (WUE) of oat. Mean of the varieties and standard errors (2021, Debrecen).

Treatments	WUE (kg m^−3^) 27 May	WUE (kg m^−3^) 10 June	WUE (kg m^−3^) 24 June
Control	5.27 ± 0.049 a	10.55 ± 0.166 a	2.37 ± 0.145 a
Si	5.75 ± 0.042 b	12.14 ± 0.152 b	7.01 ± 0.176 b
S	6.32 ± 0.051 c	15.19 ± 0.167 c	8.82 ± 0.177 c
Si + S	6.25 ± 0.049 c	14.41 ± 0.160 d	6.83 ± 0.168 d

Different letters mean statistically different values in the columns at *p* = 5%.

**Table 3:** Water use efficiency (WUE) of oat varieties. Mean of the treatments and standard errors (2021, Debrecen).

Varieties	WUE (kg m^−3^) 27 May	WUE (kg m^−3^) 10 June	WUE (kg m^−3^) 24 June
Mv Hópehely	6.32 ± 0.059 a	12.67 ± 0.199 a	5.61 ± 0.201 a
Mv Kincsem	6.27 ± 0.059 a	11.83 ± 0.199 b	5.28 ± 0.201 b
Mv Imperiál	5.87 ± 0.051 b	12.39 ± 0.173 c	7.26 ± 0.174 c
Mv Istráng	5.92 ± 0.059 c	12.38 ± 0.198 c	6.46 ± 0.201 d
GK Arany	5.75 ± 0.058 d	14.67 ± 0.195 d	7.55 ± 0.198 e
GK Impala	6.30 ± 0.072 a	14.51 ± 0.244 d	6.88 ± 0.247 f

Different lower-case letters mean statistically different values in the columns at *p* = 5%.

**Table 4:** Yield and thousand kernel weight of winter oat as an effect of the treatments (Debrecen, 2021).

Treatments	Control		Si		S		Si + S	
Varieties	Yield (kg ha^−1^)	TKW (g)	Yield (kg ha^−1^)	TKW (g)	Yield (kg ha^−1^)	TKW (g)	Yield (kg ha^−1^)	TKW (g)
GK Arany	6662.5 a	32.0 a	7331.8 a	34.7 a	7041.5 a	31.7 a	7026.8 a	34.7 a
GK Impala	5593.0 b	26.3 b	6323.3 b	24.3 b	6228.6 b	21.3 b	6750.8 b	23.7 b
Mv Hópehely	6421.6 c	35.0 c	6469.2 b	34.3 a	6718.7 c	32.7 a	6366.1 c	33.7 a
Mv Imperiál	5487.0 b	26.3 b	6523.7 b	28.0 c	6198.2 b	27.0 c	6470.0 c	30.3 c
Mv Istráng	6747.9 a	33.0 a	6821.7 c	34.3 a	6382.8 b	31.3 a	6757.3 b	34.0 a
Mv Kincsem	5112.2 d	27.0 b	6222.2 b	29.7 c	5807.7 d	29.0 c	5520.5 d	32.0 c

TKW: thousand kernel weight (g). Different lower-case letters mean statistically different values in the columns at *p* = 5%.

**Table 5:** Soil analysis results for the experimental site (2021, Debrecen).

	Layer 0–20 cm	Layer 20–40 cm	Layer 40–60 cm
pH (H_2_O)	8.30	8.36	8.43
K_A_	38	38	38
CaCO_3_ (%)	8.1	8.1	8.1
Humus (%)	3.66	2.92	2.70
NO_3_ + NO_2_ (mg kg^−1^)	1.71	2.95	3.18
NH_4_ (mg kg^−1^)	0.836	1.023	3.18
P_2_O_5_ (AL) (mg kg^−1^)	1671.6	1376.1	1076.8
K_2_O (AL) (mg kg^−1^)	658.9	648.2	525.5
SO_4_ (mg kg^−1^)	3.07	6.00	7.81

Note: K_A_: Arany-type plasticity; AL: ammonium lactate-soluble.
